# Epidemiologic trends in chronic renal replacement therapy over forty years: A Swiss dialysis experience

**DOI:** 10.1186/1471-2369-13-52

**Published:** 2012-07-02

**Authors:** Petra Rhyn Lehmann, Manon Ambühl, Domenica Corleto, Richard Klaghofer, Patrice M Ambühl

**Affiliations:** 1 Renal division, Stadtspital Waid, Zurich, Switzerland; 2 Division of Psychiatry and Psychotherapy, University Hospital Zurich and Stadtspital Waid, Zurich, Switzerland

**Keywords:** Dialysis, Epidemiology, Outcome, Time trends

## Abstract

**Background:**

Long term longitudinal data are scarce on epidemiological characteristics and patient outcomes in patients on maintenance dialysis, especially in Switzerland. We examined changes in epidemiology of patients undergoing renal replacement therapy by either hemodialysis or peritoneal dialysis over four decades.

**Methods:**

Single center retrospective study including all patients which initiated dialysis treatment for ESRD between 1970 and 2008. Analyses were performed for subgroups according to dialysis vintage, based on stratification into quartiles of date of first treatment. A multivariate model predicting death and survival time, using time-dependent Cox regression, was developed.

**Results:**

964 patients were investigated. Incident mean age progressively increased from 48 ± 14 to 64 ± 15 years from 1^st^ to 4^th^ quartile (p < 0.001), with a concomitant decrease in 3- and 5-year survival from 72.2 to 67.7%, and 64.1 to 54.8%, respectively. Nevertheless, live span continuously increased from 57 ± 13 to 74 ± 11 years (p < 0.001). Patients transplanted at least once were significantly younger at dialysis initiation, with significantly better survival, however, shortened live span vs. individuals remaining on dialysis. Among age at time of initiating dialysis therapy, sex, dialysis modality and transplant status, only transplant status is a significant independent covariate predicting death (HR: 0.10 for transplanted vs. non-transplanted patients, p = 0.001). Dialysis vintage was associated with better survival during the second vs. the first quartile (p = 0.026).

**Discussion:**

We document an increase of a predominantly elderly incident and prevalent dialysis population, with progressively shortened survival after initiation of renal replacement over four decades, and, nevertheless, a prolonged lifespan. Analysis of the data is limited by lack of information on comorbidity in the study population.

**Conclusions:**

Survival in patients on renal replacement therapy seems to be affected not only by medical and technical advances in dialysis therapy, but may mostly reflect progressively lower mortality of individuals with cardiovascular and metabolic complications, as well as a policy of accepting older and polymorbid patients for dialysis in more recent times. This is relevant to make demographic predictions in face of the ESRD epidemic nephrologists and policy makers are facing in industrialized countries.

## Background

Since intermittent renal replacement therapy has been established almost 50 years ago, the number of patients with endstage renal disease (ESRD) requiring chronic dialysis therapy has increased dramatically over the years with currently more than 2500 patients on maintenance hemodialysis therapy in Switzerland.

The first successful dialysis has been performed in a 67 year old female in 1943 with documented survival of the patient. Belding Scribner initiated the first outpatient dialysis in 1960 with a thrice weekly treatment schedule. This was the beginning of a new area of intermittent maintenance hemodialysis treatment in patients suffering from ESRD [1]. In parallel, peritoneal dialysis has evolved as another form of dialysis, using the peritoneum as semi-permeable membrane. In 1964, Robert Palmer developed a catheter for long term use [2], which was modified by Henry Tenckhoff and is still in use today.

Since the development and the implementation of chronic maintenance dialysis programs all over the world, longterm outcome of patients with endstage renal disease undergoing chronic replacement therapy, remains poor. Dialysis dose, expressed as Kt/V, urea reduction ratio (URR), nutritional status, albumin, hematocrit and body mass index have been associated with survival. Also, non-modifiable factors such as age, race, diabetes and comorbidities were exposed as modifiers of mortality and morbidity in patients on dialysis [3-6]. Similarly, regional differences in outcome have been reported [7]. Over the last four decades, many technical modifications in maintenance dialysis treatment have been realized, such as the use of disposable highflux polysulfon dialyzers and online hemodiafiltration. In addition, medical advances towards improvement in survival of patients with ESRD undergoing HD or PD have been accomplished, such as substitution therapy with human recombinant erythropoietin, and optimization of nutritional status. However, only scarce epidemiological data are available on longitudinal temporal patterns of patient and outcome characteristics on chronic renal replacement therapy, especially in Switzerland.

The aim of the present study was to analyze changes in epidemiologic characteristics and outcomes of patients undergoing renal replacement therapy by either hemodialysis (HD) or peritoneal dialysis (PD) over the last four decades in a single center in Zurich, Switzerland. Specifically, we wanted to test the hypothesis, that outcome of individuals initiated on dialysis therapy has improved with time. To this aim, we evaluated changes in mortality of patients over time in relation to patient characteristics, such as age, sex, therapy modality, time on dialysis, and transplant status.

## Methods

In this single center retrospective study all patients were included initiating treatment for ESRD either by thrice weekly hemodialysis or peritoneal dialysis in our center of the Stadtspital Waid (SWZ) hospital, Zurich, Switzerland, since start of the program in June 1970 through October 2008, and a follow-up until December 31 2010. The SWZ is one of three county hospitals with a hospital based dialysis unit in the city of Zurich, and a referral population of approximately 300'000 in the greater Zurich area. Patients are almost exclusively adults. Patients commencing renal replacement therapy in our unit were recorded by name, date of birth and therapy modality. Various hemodialysis modalities (in-center HD, home HD, limited care HD) were documented separately, but combined to one category for most analyses due to the small proportion of patients performing either home or limited care HD (Tab. 1). Moreover, changes in therapy modality, including date of renal transplantation, if applicable, were registered. Finally, date of death, if reported, was entered into the registry. Missing data were completed by exploring internal hospital records, census records of the city of Zurich as well as of surrounding counties, and by gathering additional information from practicing nephrologists in the greater Zurich area.

Overall, the resulting study population totaled 964 patients. Out of these, data were incomplete for 122 patients regarding date of birth. In 127 patients (7.6%), vital status at the end of follow-up as per December 31, 2010, was not accounted for. Patients switching between dialysis modalities were analyzed according to their initial therapy modality. In contrast, patients who were transplanted during the time of observation were analyzed separately according to initial dialysis modality and according to transplant status. Survival was analyzed as time from dialysis initiation to the end of observation at December 31^st^ 2010, or to date of death. Patients were analyzed in quartiles according to the year of renal replacement initiation (1^st^ quartile/group 1: June 1970 through August 1979; 2^nd^ quartile/ group 2: September 1979 through June 1990; 3^rd^ quartile/group 3: July 1990 through February 1999; and, 4^th^ quartile/group 4: March 1999 through December 2010).

Gathering, analysis and publication of retrospective data of patients from institutions and/or by authors (P.M.A.) affiliated with the University of Zurich (UZH) are granted a general waiver by the ethics committee of the UZH by which the present study is covered.

### Statistical analyses

In general, analyses reporting on age, sex, therapy modality and transplant status are given for an incident population commencing dialysis therapy. Descriptive statistics are given as mean ± standard deviation, percentages and confidence intervals (CI). Comparisons between groups were made by ANOVA for continuous variables and chi-square tests and Fisher’s exact test for categorial variables, respectively. We used Kaplan-Meier method to calculate survival times from date of dialysis initiation to date of death (uncensored) or to the end of observation at December 31^st^ 2010 (censored). Breslow test was calculated to compare survival times among quartiles of dialysis initiation (decades). Cox regression analysis was performed with survival time as dependent variable, status variable death (yes = 1), and quartile of dialysis initiation (decade, categorial with first quartile as reference), sex, age at dialysis initiation, dialysis modality (HD/PD) and transplant status as independent variables. We used Schoenfeld residuals test to evaluate the proportional hazard assumption. As this assumption is violated, we carried out a time dependent Cox regression analysis adding the interaction terms between time and independent variables as covariates to the Cox model above. All statistical tests are two-sided and P ≤ 0.05 was considered to be significant. All analyses were carried out with IBM SPSS Statistics, version 20 (SPSS Inc., Chicago, IL, USA) and Stata/SE 12.0 for Windows (StataCorp, College Station, Texas, USA).

## Results

From June 8, 1970 through October 24, 2008, 964 patients were enrolled at our center for maintenance dialysis due to ESRD, and, therefore, analyzed in this study. Missing data were most prevalent during the first and second decade (32 and 18%, respectively), with complete data sets for the third and fourth decade.

In Table 1, characteristics of incident patients at start of dialysis are summarized. Overall mean age was 55.0 ± 16 years, with a range from 14.7 to 89.2 years. Women were significantly older at time of first dialysis. The vast majority of patients initiated chronic replacement therapy on hemodialysis, whereas a minute proportion of participants performed limited care HD (LC) or home HD (HHD). The percentage of patients who were initiated on peritoneal dialysis was 16.8. Patients on home HD were younger, whereas those treated with LC were older compared to those on in-center HD or PD. Based on the low numbers of HHD an LC patients, however, the differences were not statistically significant. The percentage of patients performing PD was comparable between women and men (18.7 vs. 15.7%; p = 0.131). Overall, 182 patients (18.9%) were noticed for at least 1 change in dialysis modality. Of those, 43 (23.6%) switched from their initial therapy to in-center HD, 67 (36.8%) to HHD, 42 (23.1%) to LC, and 30 (16.5%) to PD. Twenty-four subjects performed a second switch, mostly to in-center HD. A total of 371 patients (38.5%) who began renal replacement therapy in our institution were transplanted at least once during follow-up. The average age at time of first transplantation was 48.3 ± 16 years.

**Table 1:** Demographic characteristics according to quartile of dialysis vintage

	**Total**	**1**^**st**^**quartile**	**2**^**nd**^**quartile**	**3**^**rd**^**quartile**	**4**^**th**^**quartile**	**P**
Age at initiation of dialysis, yr	55.0 ± 16	48.0 ± 14	49.7 ± 16	55.1 ± 15	63.9 ± 15	<0.001
Sex, %						0.138
→ Male	60.5	55.7	58.1	63.5	64.7	
→ Female	39.5	44.3	41.9	36.5	35.3	
Initial dialysis modality, %						<0.001
→ HD	80.1	92.8	52.5	78.7	96.3	
→ HHD	2.0	4.3	3.4	0	0.4	
→ PD	16.8	3.0	43.3	17.4	3.3	
→ LC	1.2	0	0.8	3.7	0	
Vital status, %						<0.001
→ Alive	23.4	2.5	19.1	29.0	43.2	
→ Dead	63.4	64.3	68.5	65.6	55.2	
→ Unknown	13.2	33.2	12.4	5.4	1.7	
Age at death, yr	65.9 ± 13	56.7 ± 13	63.2 ± 11	67.4 ± 11	73.9 ± 11	<0.001
Survival, %						
→ 3 years	71.1	64.0	72.2	77.9	67.7	
→ 5 years	60.4	52.7	64.1	66.4	54.8	

Of the subjects with certified vital status 73.0% have died during follow-up. Mean age at time of death is 65.9 ± 13 years. The average survival time censored for end of study period of patients on renal replacement therapy from first dialysis to death is 6.2 ± 6 years. Survival time after start of dialysis is 8.4 ± 8 years. Three- and five-year survival is 71.1 and 60.4%, respectively. Survival is similar for men and women (73.0 and 68.3%, respectively, for 3 yr survival (p = 0.153), and 63.1 and 56.6%, respectively, for 5 yr survival (p = 0.080)). Similarly, no significant differences in survival were found between HD and PD (data not shown).

**Figure 1:**
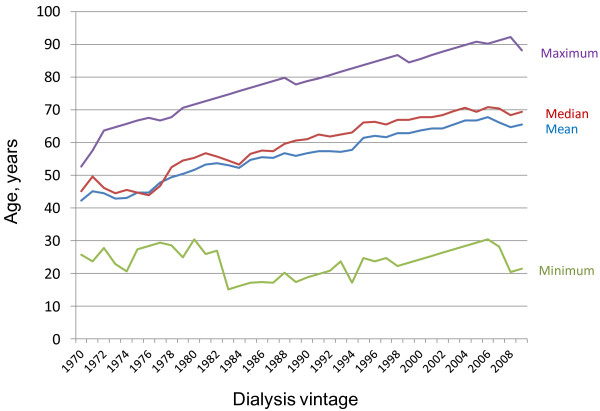
**Development of prevalent age on dialysis over 4 decades (1970–2008).** Prevalent age is given over time for every year since 1970, with separate lines for mean, median, minimal and maximal age. Only patients being on dialysis in the respective year are accounted for, excluding transplanted individuals.

In order to analyze time trends patients were subdivided into quartiles regarding dialysis vintage, resulting in groups of approximately 10 year intervals (“decades”). Significant differences can be noticed between the four groups with regard to incident patients’ age at time of dialysis initiation (Table 1). Over the observation period of almost 40 years a gradual increase in mean incident age from 48.0 (median: 48.4) to 63.9 (median: 63.6) years can be observed (Table 1). The ratio of men to women within groups was 1.13, 1.46, 1.74, and 1.84 for the 1^st^, 2^nd^, 3^rd^, and 4^th^ decade, respectively, demonstrating a continuously developing predominance of males starting renal replacement therapy in our cohort. Figure 1 depicts development in prevalent age of the entire cohort being on dialysis treatment over 40 years, with a continuous rise both in mean and median age of the population. Half of the population was beyond 60 years in 1989, and older than 70 in 2004. Age was higher among women in the overall population, but only during the first two decades of dialysis initiation (data not shown). A significant drop in incident home dialysis patients, both PD and home HD, can be observed, from a peak 47 percent in the 2^nd^ quartile to less than 4 percent in the new millennium (Table 1).

**Figure 2:**
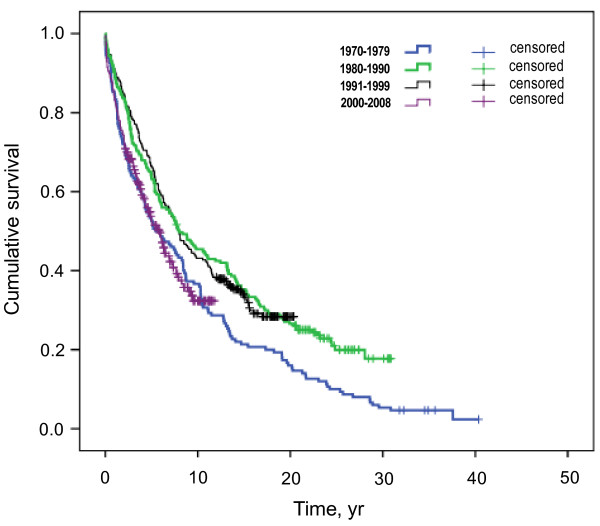
**Kaplan-Meier survival curve according to dialysis vintage.** Cumulative survival by Kaplan-Meier analysis is shown separately for each quartile of patients according to dialysis vintage between 1970 and 2008 (Breslow test: chi-square = 16.75, df = 3, p = 0.001).

**Figure 3:**
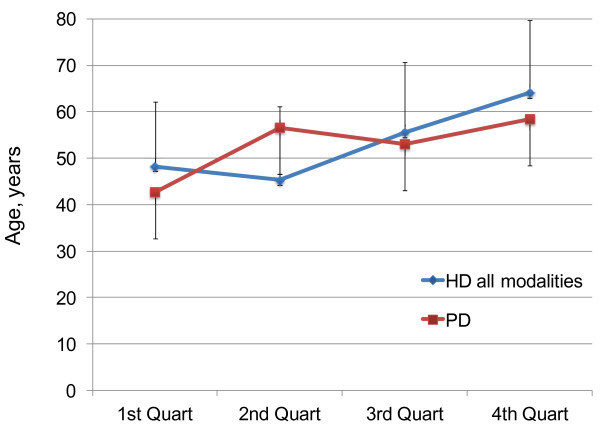
**Age distribution between hemodialysis (HD) and peritoneal dialysis (PD) patients over 4 decades.** Incident age for the respective quartiles of dialysis vintage is shown for HD and PD patients. Results are presented as mean age at time of therapy initiation (symbols) and standard deviation (error bars). Whereas for PD patients incident age remained relatively stable over years, a continuous increase for patients on HD can be noted. However, differences are not statistically significant at any time point between dialysis modalities.

Regarding the age at time of death, significant differences are documented between the four groups, with increasing mean age at initiation of dialysis therapy over time from 56.7 ± 13 yr in group 1 to 73.9 ± 11 yr in group 4 (Table 1). In contrast, mean survival time to death of patients on renal replacement therapy from onset of dialysis therapy significantly decreased from 8.3 years in the most ancient group to 2.9 years in the quartile of patients initiating renal replacement most recently. Figure 2 reveals the lowest cumulative survival for patients of the first and forth quartile. In addition to survival characteristics, we sought for other factors distinguishing subgroups stratified to dialysis vintage. With regard to therapy modality, patients initiating on peritoneal dialysis have a more homogenous age distribution over time vs. patients on hemodialysis (Figure 3).

**Figure 4:**
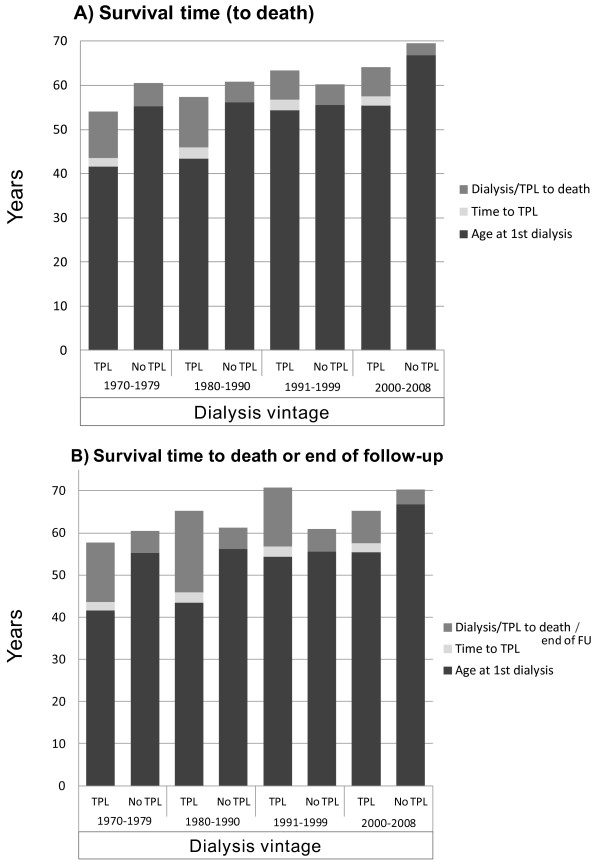
**Time trends in renal replacement therapy over 4 decades.** Columns represent mean age at time of first dialysis and death, and mean duration from first dialysis to transplantation (if applicable) and to death (**A**) or to death/end of follow-up (**B**) in years. Columns are differentiated according to dialysis vintage and transplant status. For statistical characteristics among groups refer to Table 2.

Furthermore, we analyzed differences between transplanted vs. non-transplanted patients as well as time trends in transplantation and their effects on patient survival (Table 2). A total of 371 patients (38.5%) received a kidney transplant within the observation period of 40 years. Men were significantly more likely to receive a transplant than women (43.3 vs. 31.5%, respectively; p < 0.001). The number of transplanted individuals was comparable within the first, second and third time interval with 94, 111, and 102 transplants performed, respectively. During the most recent decade, however, the number of transplantations decreased significantly by 40 percent (p < 0.001). Inversely, mean age at transplantation continuously and significantly increased from 45 to 53 years over time (Table 2). Patients undergoing transplantation were significantly younger at time of dialysis onset compared to those not being transplanted during follow-up. Analyzed by subgroups according to dialysis vintage similar findings were noticed for all 4 time periods, except for the 3^rd^ decade, when age at transplantation increased by 11 years compared to the preceding time period (see Figure 4). Surprisingly, waiting time to first transplantation after start of dialysis increased only slightly from 27 to 29.8 months from the 1^st^ to the 4^th^ decade.

**Table 2:** Demographic characteristics for transplanted vs. not transplanted patients (all patients and patients stratified according to quartile of dialysis vintage)

**Dialysis vintage, quartile**	**Transplanted**	**Not transplanted**
	**All pts. / 1**^**st**^**/ 2**^**nd**^**/ 3**^**rd**^**/ 4**^**th**^	**All pts. / 1**^**st**^**/ 2**^**nd**^**/ 3**^**rd**^**/ 4**^**th**^
Age at 1^st^ dialysis, yr	**48.3 ± 16**^¥^ / 41.6 ± 11 / 43.4 ± 15 /54.4 ± 16 / 55.4 ± 16 ^†^	**59.7 ± 15** / 55.3 ± 13 / 56.1 ± 14 / 55.6 ± 15 / 66.8 ± 14 ^†/¶^
Time to TPL, yr	**2.0 ± 3**^¥^ / 2.5 ± 3 / 2.3 ± 3 / 2.2 ± 4 ^§^	--
Age at TPL, yr	**45.6 ± 14**^¥^ / 44.7 ± 11 / 44.5 ± 14 / 47.8 ± 14 / 52.7 ± 16 ^†^	--
Age at death, yr	**58.5 ± 13**^¥^ / 54.5 ± 13 / 60.4 ± 10 / 62.8 ± 10 / 59.2 ± 22 *	**68.8 ± 12** / 59.9 ± 11 / 64.6 ± 11 /68.8 ± 11 / 74.6 ± 10 ^†^
Survival after initiation of dialysis, %		
3 years	**92.4**^¥^ / 74.3 / 95.6 / 99.0 / 98.3	**57.1** / 55.0 / 54.2 / 62.3 / 56.1 ^‡^
5 years	**87.9**^¥^ / 67.1 / 93.4 / 93.8 / 96.0	**41.5** / 40.0 / 41.5 / 46.2 / 37.3 ^‡^

¥: p < =0.001 vs. patients not transplanted.

†: p < =0.001 among quartiles.

*: p < 0.050 among quartiles.

‡: p < 0.050 for all quartiles vs. respective transplant periods.

§: p = NS.

¶: p < 0.050 for all quartiles vs. respective transplant periods except for "age at 1^st^ dialysis" in 3^rd^ quartile.

**Figure 5:**
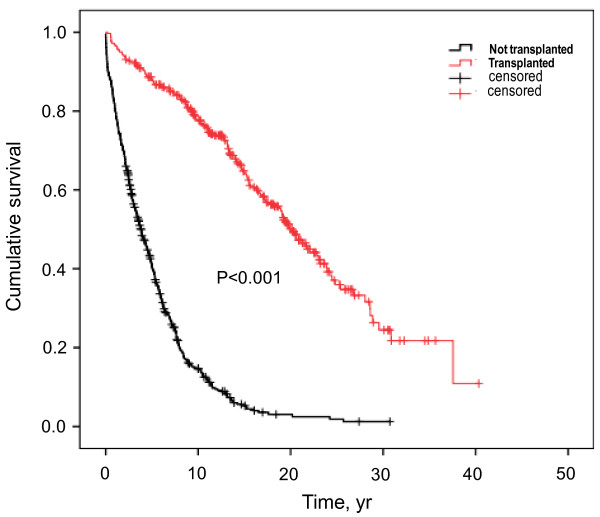
**Kaplan-Meier survival curve according to transplant status.** Kaplan-Meier curve for cumulative survival stratified for transplant status of the entire cohort (Breslow test: chi-square = 283.70, df = 1, p < 0.001).

Cumulative survival is significantly longer in transplanted versus non-transplanted individuals (Figure 5, Table 2). Accordingly, both 3- and 5-year survival is 1.6- and 2.1-fold higher in transplant versus non-transplant patients, respectively. However, this effect is time-dependent and decreasing from earlier to later stages after transplantation. The survival advantage for transplanted individuals is also apparent in subgroups stratified for decade of dialysis vintage (Table 2).

**Table 3:** Cox-regression analysis (time-dependent)

	**Hazard Ratios for death (HR)**	**95%CI for HR**		**P**
		Lower	Upper	
Dialysis initiation, 1^st^ quartile (ref.)	1			
Dialysis initiation, 2^nd^ quartile	0.68	0.48	0.96	0.026
Dialysis initiation, 3^rd^ quartile	0.82	0.55	1.22	0.320
Dialysis initiation, 4^rd^ quartile	0.95	0.61	1.47	0.803
Age at 1^st^ dialysis, yr	1.01	1.00	1.02	0.063
Sex (1: male)	1.02	0.80	1.31	0.858
HD (1) vs. PD	1.23	0.87	1.74	0.244
TPL (1: transplanted)	0.10	0.06	0.15	<0.001
**Time dependent covariates** (independent variables × time)				
Dialysis initiation, quartile	0.99	0.97	1.01	0.287
Age at 1^st^ dialysis, yr	1.00	1.00	1.00	0.275
Sex (1: male)	1.00	0.97	1.03	0.985
HD (1) vs. PD	1.01	0.97	1.05	0.592
TPL (1)	1.07	1.02	1.11	0.002

With survival time as dependent variable, status variable death (1), and quartile of dialysis initiation (decade, categorial with first quartile as reference), sex, age at dialysis initiation, dialysis modality (HD/PD) and transplant status as independent variables and time dependent covariates.

Finally, we sought to develop a model predicting survival and to examine the hypothesis that survival of dialysis patients in our cohort has improved over the last four decades. In order to account for significant Schoenfeld residuals test, we performed time-dependent Cox regression analysis with dialysis vintage (quartile), age at dialysis initiation, sex, dialysis modality (HD vs. PD) and transplant status as covariates. As shown in Table 3, only transplant status turns out to be an independent predictor of survival with a hazard ratio of 0.10 for death in transplanted versus non-transplanted individuals (p < 0.001). Moreover, a significant effect of dialysis vintage is observed only between the second and first quartile, with a hazard ratio for death of 0.68 (p = 0.026), suggesting a survival benefit during the second time interval.

## Discussion

This retrospective analysis reports on epidemiologic time trends in more than 900 patients over forty years initiating renal replacement therapy on dialysis. To our best knowledge, this is the only study being published, so far, covering a time span of this magnitude. The major findings of this evaluation are: *a)* a continuous trend of increasing age both in incident and prevalent patients over time; *b)* a predominance of male patients initiating dialysis therapy at a younger age compared to women; *c)* a continuous decrease in patients initiating renal replacement on home-based therapy regimes; *d)* a drop in survival during the most recent decade; *e)* a significant survival advantage for patients being transplanted; and, *f)* overall, outcome on dialysis is probably mostly affected by progress in the medical management of comorbidities rather than improvements in dialytic therapy per se.

During the time period of forty years, we observed an increase in age at time of starting dialysis from 48 in the 1970s to 64 years during the last decade between 1999 and 2008. This finding reflects the trend of increasing age of incident dialysis patients in developed countries as reported in the ERA-EDTA Registry Annual Report with a mean age at initiation of dialysis of 64 years in 2008, and of 70 years in Germany in 2006 [8,9]. The data of the DOPPS study from 2004 show an age range from 58.0 years in the UK to 62.4 years in Italy [10]. Also, our data correspond well with reports from Switzerland by Saudan et al. from the French part of the country [6], and by Breidthart et al. from Basel [11]. In Saudan's publication, mean age of 64 ± 15 years in prevalent patients of 2001 matched exactly that in our own cohort for the same year (Figure 1). In Breidthardt's report, mean age during the observational period between 1995 and 2006 equaled 65. Several explanations may account for this finding. First, the average age of the population in the Western world is increasing generally, and, second, improvements in medical management and survival of patients with illnesses finally resulting in endstage renal disease (ESRD) have been achieved over the last decades. For example, we have shown previously, that patients in Switzerland with type 1 diabetes mellitus (DM) undergoing renal transplantation had a gradual increase in duration from onset of diabetes to ESRD over the last five decades [12].

A predominance of male subjects among patients initiating dialysis was found in our cohort, with a continuous increase from 56 to 65% during the observation period. Moreover, men were consistently younger at time of first dialysis by about six years during the first two decades, and caught up with women only in the last ten years of our analysis. These findings may either be explained by better screening and medical treatment of male subjects in our society, or reflect higher disease burden in men. Neither hypothesis can be confirmed by our study. Of note, comparable findings were made independently by Saudan et al., as well as by Breidthardt and coworkers regarding the higher percentage of male individuals in their Swiss cohorts (63 and 56% male patients, respectively). Nevertheless, outcome is comparable for men and women in our study, with only slightly lower 3- and 5-year survival by about 8 percent each in women. This difference can easily be explained by the higher age at dialysis start in women. As a consequence, mean age at death was almost identical between sexes with 65.7 and 66.2 years for male and female subjects, respectively.

Our data clearly indicate a relevant drop in home dialysis regimens since the nineteen nineties. Whereas in the 2^nd^ quartile of our analysis nearly half of our population performed a self care based dialysis modality at home, a mere 4 percent of incident patients were on either PD or home HD since the turn of the millennium. This reflects a common trend of declining numbers in home based therapy in Switzerland. In 1985, 41% of the Swiss dialysis population was on self care treatment, with only 12.9% in 2009 [13]. Several factors have contributed to this development. With a growing number of ESRD patients and treatment possibilities restricted to a limited number of mainly hospital based hemodialysis units, many centers established PD and home HD programs, in order to keep up with the increasing demand for dialysis therapy. Over the years, however, new capacities for in-center HD were provided, especially by hemodialysis units in private nephrology cabinets outside of hospital settings. In addition, lesser reimbursement for PD in Switzerland may occasionally have favored in-center HD, especially with increasing supply of the latter. Finally, the most important reason can be explained by medical factors, as illustrated by our study. As mentioned before, incident age of the ESRD population both internationally and in our cohort has continuously increased, most dramatically in the 3^rd^ quartile (beginning in 1990). In contrast, the average age of incident PD patients remained almost stable from the 2^nd^ quartile to the end of the observation period (Figure 3). This most certainly reflects that PD is a more age related therapy modality, preferentially chosen by and adequate for patients in a younger to middle age group.

We found 3- and 5-year overall survival rates of 71 and 60 percent, respectively, in our study population. These findings are in agreement with the two recently published studies from Switzerland mentioned above with comparable socio-economic background, analyzing patient data from around the year 2000. They documented 3- and 5-year survival of 68 and 46%, respectively, in Basel [11], and 61% 3-year survival in the French part of Switzerland [6]. Survival after initiation of dialysis decreased significantly in our cohort by about 50 and 35 percent in survival time censored for study follow-up and time to death, respectively, since 1970. This drop is explained exclusively by changes occurring in the last quartile of observation between 2000 and 2008. Obviously, part of this finding is inherent to the study design with progressively shorter follow-up from earlier to later segments in the time frame under examination. However, corresponding drops in the 3- and 5-year survival in the last quartile of the cohort are indicative of a clear trend to lower survival in most recent years, which is not influenced by methodological factors. Accordingly, the shape of the Kaplan-Meier curve for cumulative survival, which is directly comparable for the first 10 years between each subgroup, is compatible with this trend. One major explanation for this development, obviously, is the increasing age at start of renal replacement therapy. The CRIB study showed the risk of death approximately to double for each 15 years of older age [14]. In the DOPPS study, Cox models adjusted for demographics and a wide range of comorbidities, showed elderly patients to have a three- to sixfold higher mortality risk compared with participants below 45 years of age [15]. Older age per se is associated with shorter survival, but is also linked to more comorbidities [14-17]. Despite poorer survival after initiation of dialysis, patients’ life span has continuously been extended from 57 yr in the first quartile of the observation period to 74 yr during the most recent decade of our analysis.

Transplantation in patients with endstage renal disease has been shown in several studies to be associated with better outcomes [18,19]. In our analysis, both 3- and 5-year survival were clearly superior in transplanted individuals compared to patients remaining on dialysis. Again, this is explained, in part, by a lead-time error with significantly lower age at initiation of dialysis therapy in patients undergoing transplantation at a later stage in life. After multivariate adjustments using time-dependent Cox regression, however, patients not being transplanted had a 10-fold higher risk for death versus non-transplanted individuals (HR of 0.10 for transplanted vs. non-transplanted patients). In addition to lower age, patients chosen for a transplant may be "better risks", selections among those with less comorbidities. However, transplantation by itself, providing higher solute clearance, better metabolic control and production of endogenous hormones, confers lower morbidity and better survival. Nevertheless, survival time of transplanted individuals has progressively dropped, especially in the 3^rd^ and 4^th^ quartile of our analysis. Despite this circumstance, 3- and 5-year patient survival was still maintained clearly beyond 90 percent. This may be indicative of the fact, that mean age at transplantation, despite a continuous increase over time, is still relatively low at 53 years. With mean survival time censored for study follow-up still being almost 8 years, its drop within the last 20 years is primarily due to the older individuals among the transplant patients. The percentage of patients older than 60 years increased from 7% in the 1^st^ to 39% in the 3^rd^ decade. Concomitantly, survival times after transplantation below and beyond 60 years of age were 11.1 and 6 years in the 1^st^, and 9.4 and 4.3 years in the 3^rd^ decade, respectively. In accordance with these findings, it has been shown that transplantation at an age beyond 65 years is not always associated with a survival benefit compared to dialysis therapy [19-23].

Apart of analyzing the changing characteristics of patients starting on renal replacement therapy over the last 40 years, one specific aim of the current study was to address the question, whether medical management of an ESRD cohort in Switzerland has improved over time, resulting in better outcomes in this population. Among general medical treatment options, development of new pharmaceutical compounds, such as recombinant human erythropoietin, as well as better care of patients with diabetes and cardiovascular complications can be considered to have beneficial effects on morbidity and mortality. Along with these achievements, technical and procedural innovations in dialysis therapy may have translated into improvements in patient survival. In order to examine this hypothesis, we used the epidemiological characteristics of our cohort under investigation to develop a model predicting death or survival time by stratification into quartiles of dialysis vintage and adjusting for potential confounders such as age, sex, initial dialysis modality, transplant status and follow-up time. However, except for transplant status, none of the other covariates were predictive of the defined outcome. Conversely, later dialysis vintage was noted to confer a survival benefit only for the second versus the first quartile. These findings suggest, that reaching progressively older age on renal replacement therapy over the last 40 years is not directly and exclusively related to technological advances in dialysis therapy and medical treatment of patients with endstage renal disease. Improvements in general medical care may have contributed indirectly, by prolonging the life of patients with diabetes mellitus and cardiovascular diseases, both pharmaceutically and through interventional techniques like coronary artery bypass surgery and endovascular procedures for cardiac, cerebral and peripheral vasculopathies. Moreover, the increasing proportion of elderly people may be explained by policies accepting older and polymorbid patients for renal replacement therapy in the last 10 to 20 years. As a result, an increasing number of older patients experience secondary renal failure from systemic illness and/or age, rather than dying from their primary disease. Once on dialysis, however, prognosis is limited by their comorbid conditions and age. This will impact on future developments of treatment numbers of patients being on dialysis. As incident cases may continue to increase, prevalent patients probably will reach a plateau in the near future due to the dynamics described in our analysis.

The present study has several limitations. First, it was performed retrospectively, thus limiting the accuracy in data collection and the possibilities for correction of potential confounders. Also, despite a fairly high overall completeness of more than 90% in gathered records, data were missing in almost one third of patients from the first decade of the study period. However, there are no indications of a systematic lack of information. Moreover, analyses like this, with a time span covering 40 years, are impossible to carry out in a prospective manner, for obvious reasons. Second, our analysis is based on a single center experience. This prohibits generalizations to other settings. Nevertheless, based on comparisons with published data from other Swiss centers, we are fairly confident, that our results are representative for Switzerland and many other central European countries with comparable health care systems and socioeconomic characteristics. Third, our study cannot compete with much larger registries such as the United States renal data system (USRDS) or the ERA-EDTA registry. However, for Switzerland no national ESRD data sources are available, with the exception of a Swiss registry that has been implemented only in 2006. Fourth, no data about the diagnosis causing endstage renal disease, and further detailed information on comorbidity, nutritional status, quality of dialysis, and laboratory results in our patients are available. Nevertheless, we have no reason to assume that our cohort differs significantly from that of other institutions in Switzerland with regard to epidemiological and medical characteristics. This is supported by many similarities derived from other studies mentioned before.

In conclusion, our analysis of 40 years of single center dialysis experience provides relevant information on epidemiologic trends in changing characteristics of the ESRD population in Switzerland, as well as on policies in the implementation of renal replacement therapy. Our findings document an increase of a predominantly elderly incident and prevalent dialysis population with shortened survival after initiation of renal replacement, and, nevertheless, prolonged lifespan. The latter seems not exclusively attributable to medical and technical advances in dialysis therapy, but rather to reflect better survival of individuals with cardiovascular and metabolic complications, as well as a policy of accepting older and polymorbid patients for dialysis. These findings are relevant to predict future developments in face of the ESRD epidemic nephrologists and policy makers are facing in industrialized countries.

## Competing interests

None of the authors declares any competing interests.

## Authors’ contributions

PRL, MA and DC were involved in data collection and processing, as well as outcome assessment and validation. PRL, in addition, wrote the first draft of the manuscript. RK was the statistical advisor and performed special statistical analyses. PMA initiated the project, developed the study design, supervised data collection, performed statistical analyses of the data, and wrote the final draft of the manuscript. All authors read and approved the final version of the manuscript.

## References

[B1] Cameron J History of the treatment of renal failure by dialysis 2002 Oxford University Press, New York

[B2] Palmer RA Quinton WE Gray JE Prolonged Peritoneal Dialysis for Chronic Renal Failure Lancet 1964 1 700 2 14107960

[B3] Cheung AK Levin NW Greene T Agodoa L Bailey J Beck G Clark W Levey AS Leypoldt JK Ornt DB Rocco MV Schulman G Schwab S Teehan B Eknoyan G Effects of high-flux hemodialysis on clinical outcomes: results of the HEMO study J Am Soc Nephrol 2003 14 3251 63 14638924 10.1097/01.asn.0000096373.13406.94

[B4] Port FK Ashby VB Dhingra RK Roys EC Wolfe RA Dialysis dose and body mass index are strongly associated with survival in hemodialysis patients J Am Soc Nephrol 2002 13 1061 6 11912267 10.1681/ASN.V1341061

[B5] Sanabria M Munoz J Trillos C Hernandez G Latorre C Diaz CS Murad S Rodriguez K Rivera A Amador A Ardila F Caicedo A Camargo D Diaz A Gonzalez J Leguizamon H Lopera P Marin L Nieto I Vargas E Dialysis outcomes in Colombia (DOC) study: a comparison of patient survival on peritoneal dialysis vs hemodialysis in Colombia Kidney Int Suppl 2008 S165 72 18379541 10.1038/sj.ki.5002619

[B6] Saudan P Kossovsky M Halabi G Martin PY Perneger TV Quality of care and survival of haemodialysed patients in western Switzerland Nephrol Dial Transplant 2008 23 1975 81 18156654 10.1093/ndt/gfm915

[B7] Held PJ Brunner F Odaka M Garcia JR Port FK Gaylin DS Five-year survival for end-stage renal disease patients in the United States, Europe, and Japan, 1982 to 1987 Am J Kidney Dis 1990 15 451 7 2333867 10.1016/s0272-6386(12)70363-3

[B8] Frei U Schober-Halstenberg H Nierenersatztherapie in Deutschland Quasi-Niere 2008

[B9] Stel VS van de Luijtgaarden MW Wanner C Jager KJ The 2008 ERA-EDTA Registry Annual Report-a precis NDT Plus 2011 4 1 13 21245934 10.1093/ndtplus/sfq191 PMC3022422

[B10] Rayner HC Pisoni RL Bommer J Canaud B Hecking E Locatelli F Piera L Bragg-Gresham JL Feldman HI Goodkin DA Gillespie B Wolfe RA Held PJ Port FK Mortality and hospitalization in haemodialysis patients in five European countries: results from the Dialysis Outcomes and Practice Patterns Study (DOPPS) Nephrol Dial Transplant 2004 19 108 20 14671046 10.1093/ndt/gfg483

[B11] Breidthardt T Moser-Bucher CN Praehauser C Garzoni D Bachler K Steiger J Dickenmann M Mayr M Morbidity and mortality on chronic haemodialysis: a 10-year Swiss single centre analysis Swiss Med Wkly 2011 141 w13150 21328099 10.4414/smw.2011.13150

[B12] Cao C Hellermann JP Weber M Ambühl PM Time trends in the epidemiology of renal transplant patients with type 1 diabetes mellitus over the last four decades Nephrol Dial Transplant 2006 21 770 5 16401627 10.1093/ndt/gfi278

[B13] Report of the Swiss insurance companies on renal replacement therapy in Switzerland 2010

[B14] Landray MJ Emberson JR Blackwell L Dasgupta T Zakeri R Morgan MD Ferro CJ Vickery S Ayrton P Nair D Dalton RN Lamb EJ Baigent C Townend JN Wheeler DC Prediction of ESRD and death among people with CKD: the Chronic Renal Impairment in Birmingham (CRIB) prospective cohort study Am J Kidney Dis 2010 56 1082 94 21035932 10.1053/j.ajkd.2010.07.016 PMC2991589

[B15] Canaud B Tong L Tentori F Akiba T Karaboyas A Gillespie B Akizawa T Pisoni RL Bommer J Port FK Clinical practices and outcomes in elderly hemodialysis patients: results from the Dialysis Outcomes and Practice Patterns Study (DOPPS) Clin J Am Soc Nephrol 2011 6 1651 62 21734085 10.2215/CJN.03530410 PMC4204107

[B16] Lowrie EG Huang WH Lew NL Death risk predictors among peritoneal dialysis and hemodialysis patients: a preliminary comparison Am J Kidney Dis 1995 26 220 8 7611256 10.1016/0272-6386(95)90177-9

[B17] Wagner M Ansell D Kent DM Griffith JL Naimark D Wanner C Tangri N Predicting mortality in incident dialysis patients: an analysis of the United Kingdom Renal Registry Am J Kidney Dis 2011 57 894 902 21489668 10.1053/j.ajkd.2010.12.023 PMC3100445

[B18] Schnuelle P Lorenz D Trede M Van Der Woude FJ Impact of renal cadaveric transplantation on survival in end-stage renal failure: evidence for reduced mortality risk compared with hemodialysis during long-term follow-up J Am Soc Nephrol 1998 9 2135 41 9808102 10.1681/ASN.V9112135

[B19] Wolfe RA Ashby VB Milford EL Ojo AO Ettenger RE Agodoa LY Held PJ Port FK Comparison of mortality in all patients on dialysis, patients on dialysis awaiting transplantation, and recipients of a first cadaveric transplant N Engl J Med 1999 341 1725 30 10580071 10.1056/NEJM199912023412303

[B20] Foley DP Patton PR Meier-Kriesche HU Li Q Shenkman B Fujita S Reed A Hemming AW Kim RD Howard RJ Long-term outcomes of kidney transplantation in recipients 60 years of age and older at the University of Florida Clin Transpl 2005 16 101 9 17424728

[B21] Oniscu GC Brown H Forsythe JL How old is old for transplantation? Am J Transplant 2004 4 2067 74 15575911 10.1111/j.1600-6143.2004.00622.x

[B22] Oniscu GC Brown H Forsythe JL How great is the survival advantage of transplantation over dialysis in elderly patients? Nephrol Dial Transplant 2004 19 945 51 15031354 10.1093/ndt/gfh022

[B23] Oniscu GC Brown H Forsythe JL Wolfe RA Ashby VB Milford EL Ojo AO Ettenger RE Agodoa LY Held PJ Port FK Schnuelle P Lorenz D Trede M Van Der Woude FJ Impact of cadaveric renal transplantation on survival in patients listed for transplantation. Comparison of mortality in all patients on dialysis, patients on dialysis awaiting transplantation, and recipients of a first cadaveric transplant J Am Soc Nephrol 2005 16 1859 65 15857921 10.1681/ASN.2004121092

